# Gene transfer and genome editing for familial hypercholesterolemia

**DOI:** 10.3389/fmmed.2023.1140997

**Published:** 2023-04-03

**Authors:** Cesare Canepari, Alessio Cantore

**Affiliations:** ^1^ San Raffaele Telethon Institute for Gene Therapy (SR-Tiget), IRCCS San Raffaele Scientific Institute, Milan, Italy; ^2^ Vita-Salute San Raffaele University, Milan, Italy

**Keywords:** gene therapy, familial hypercholesterolemia, gene editing, lipoproteins, lipid-lowering medications

## Abstract

Familial hypercholesterolemia (FH) is an autosomal dominant inherited disease characterized by high circulating low-density lipoprotein (LDL) cholesterol. High circulating LDL cholesterol in FH is due to dysfunctional LDL receptors, and is mainly expressed by hepatocytes. Affected patients rapidly develop atherosclerosis, potentially leading to myocardial infarction and death within the third decade of life if left untreated. Here, we introduce the disease pathogenesis and available treatment options. We highlight different possible targets of therapeutic intervention. We then review different gene therapy strategies currently under development, which may become novel therapeutic options in the future, and discuss their advantages and disadvantages. Finally, we briefly outline the potential applications of some of these strategies for the more common acquired hypercholesterolemia disease.

## Introduction

Dyslipidemias can arise from a broad spectrum of causes. They can be divided into familial dyslipidemias, in which the genetic causes are well defined, and acquired dyslipidemias, which are multifactorial. In the latter case, indeed, dietary or unhealthy behavioral habits (junk food, lack of exercise, and smoking) can be identified as contributing causes, but dyslipidemia can even develop as a bystander effect of diabetes or aging. This review focuses on familial hypercholesterolemia (FH) and on attempts to cure it over the years, from the introduction of statins through the introduction of gene therapies. Despite excellent results obtained, especially over the last few years, the optimal balance of the lipid profile remains to be fully achieved. This is particularly true in homozygous familial hypercholesterolemia (HoFH) patients, who require the strongest decrease in circulating low-density lipoproteins (LDLs). Compared to heterozygous FH (HeFH) patients and people with cardiovascular disease (CVD) unrelated to genetic mutations, HoFH patients represent the category that benefits least from most of the available drugs. This review starts with a general description of lipoprotein metabolism in a physiological condition, focusing on the two main organs responsible for fats and cholesterol balance: the liver and the intestine.

### Lipoprotein processes

#### Dietary lipids and cholesterol absorption

**FIGURE 1:**
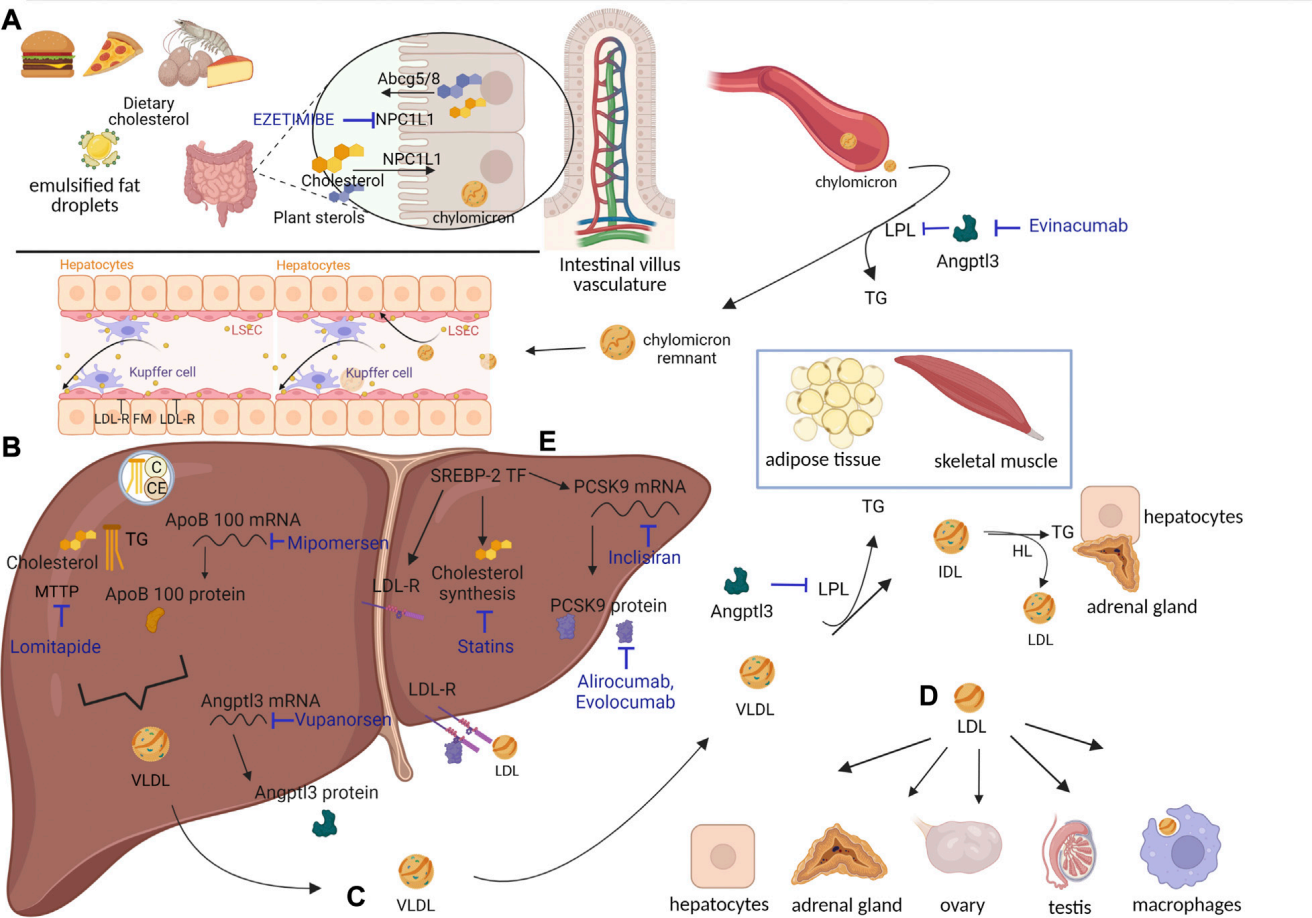
Lipoprotein metabolic processes and cholesterol-lowering drugs. **(A)** Cholesterol assumed from diet reaches enterocytes through NPC1L1. There, cholesterol esters (CEs) and triglycerides (TGs) are packaged into chylomicrons (CMs). Once in circulation, CMs are deprived of most of their TGs, which are delivered to adipose tissue and skeletal muscle through the action of lipoprotein lipases (LPLs). CMs turn into CM remnants, which access hepatocytes through LDL-R and LDL-R family members (FMs). **(B)** Cholesterol contained in CM remnants can be used by hepatocytes or packaged in VLDL and secreted into circulation. SREBP-2 transcription factor is responsible for synthesis of i) cholesterol, ii) LDL-R, and iii) PCSK9 (see also Figure 2). **(C)** VLDLs are deprived of most TGs, delivered to adipose tissue and skeletal muscle through action of LPL, and turned into IDL. IDL can either access hepatocytes through LDL-R and LDL-R FM or be converted into LDL by hepatic lipases (HLs). **(D)** LDL can be internalized by hepatocytes or move to districts particularly dependent on cholesterol for hormonal synthesis, like adrenal glands and gonads. LDL can also be phagocytosed by macrophages, which, in the case of LDL overload, appear as foam cells. Cholesterol-lowering drugs, inhibiting different targets, have been developed (written in blue). Specifically, ezetimibe inhibits NPC1L1, evinacumab inhibits ANGPTL3, mipomersen inhibits ApoB100 (at RNA level), lomitapide inhibits MTTP, vupanorsen inhibits ANGPTL3 (at RNA level), inclisiran inhibits PCSK9 (at the RNA level), alirocumab–evolocumab inhibits PCSK9, and statins inhibit cholesterol synthesis. Created with BioRender.com. **(E)** SREBP-2 transcription factor induces cholesterol synthesis and the expression of LDL-R and PCSK9.

The digestion of lipids—triglycerides (TGs), cholesterol esters (CEs), and phospholipids (PLs)—assumed through the diet starts in the oral cavity and is mediated by lipases produced by the salivary glands. It continues in the stomach, where it is mediated by the action of gastric lipases. Lipids are then subjected to hydrolysis and micellization in the duodenum. Pancreatic lipases break down TG-generating 2-monoacylglycerol (MAG) and two molecules of free fatty acids (FFAs) (Xiao et al., 2019). Phospholipase A2 and CE hydrolase act on PLs, generating FFA and lysophospholipids, and on CEs, generating cholesterol and FFA. These forms are required to access enterocytes. FFAs and PLs should be absorbed mainly through passive diffusion. In contrast, cholesterol requires active transport, which is mediated by Niemann–Pick C1-like protein 1 (NPC1L1) (Altmann et al., 2004) (Figure 1A). NPC1L1 is a target for lowering cholesterol levels (via ezetimibe, discussed in the following section). In addition to NPC1L1, ATP-binding cassette subfamily G members 5 (ABCG5) and 8 (ABCG8) are involved in cholesterol transport. They function as a heterodimer and are responsible for the opposite function as NPC1L1: the transport of sterols from the enterocytes back into the intestinal lumen. They are highly specific to plant-derived sterols. ABCG5 and ABCG8 transport, together with plant sterols, even cholesterol when it is in excess. However, less than 5% of dietary plant sterols are absorbed as compared to 45%–55% of cholesterol (Yu et al., 2002a). In the enterocytes, TGs and CEs are re-synthesized in the smooth endoplasmic reticulum (ER). Since cholesterol and TGs are hydrophobic molecules, they must be assembled into a particle, called lipoprotein, each time they travel in the circulation. Lipoproteins are made of CEs and TGs surrounded by free cholesterol, PLs, and apolipoproteins (Xiao et al., 2019). All lipoproteins rely on apolipoprotein B (ApoB) as a structural component. Particularly, the lipoproteins generated in enterocytes are called chylomicrons (CMs). Their assembly occurs through the action of chaperone microsomal TG transfer protein (MTTP). As expected, MTTP expression is enriched at the level of the villus, the highest absorbing region, but is almost absent at the level of the crypt under a standard diet (Swift et al., 2005). MTTP, located in the lumen of mammalian intestinal and hepatic microsomes, can make the protein coil around TG and CE during ApoB translation, incorporating them into a particle. Only one molecule of ApoB is required *per* lipoprotein (Sniderman et al., 2019). ApoB contains 4536 residues in its complete form, ApoB100. However, since its transcript is subjected to the action of the RNA-editing enzyme complex, which in humans is active in the intestine, the structural component of CM is ApoB48, comprising the first 2152 residues, where “48” stands for 48% of the ApoB100 form (Chen et al., 1987; Powell et al., 1987). The molecular mechanism responsible for producing ApoB48 is a site-specific RNA modification: C–U RNA editing mediated by ApoB mRNA-editing enzyme catalytic polypeptide 1 [APOBEC 1 (Swift et al., 2005)]. CMs are secreted by enterocytes at the basolateral membrane and enter the lamina propria. After traveling through lymphatic ducts, they access the circulation at the level of the left subclavian vein. There, several lipoproteins bind to CMs. The apolipoproteins which are transferred to CMs, once in the blood, are exchangeable apolipoproteins. Indeed, they are transferred over time among lipoproteins, including ApoCII, ApoAV, and ApoE. They derive from high-density lipoproteins (HDLs, discussed in more detail in the following sections) and are essential for the rest of the CM journey (Mehta and Shapiro, 2022).

#### Distribution of TG to peripheral tissues by CM

Once in circulation, CMs first travel to peripheral capillary beds of adipose tissues and skeletal muscles, which are the tissues in the greatest need of TGs—to be used for energy or lipogenesis—which the CMs are carrying. This process is mediated by lipoprotein lipases (LPLs) exposed on the lumens of these vessels. TG lipases are a family comprising LPL, endothelial lipases (LIPGs), and hepatic lipases (HLs) (Mehta and Shapiro, 2022). Lipases are responsible for the hydrolysis of TGs present in a lipoprotein into FFA and glycerol and for their subsequent uptake by tissues. As mentioned previously, once in circulation, CMs are decorated with several apolipoproteins, whose functions are defined as follows:

i) ApoAV is involved in the docking of CM on the endothelial wall, by interacting with heparan sulfate proteoglycans (HSPGs) present there (Nilsson et al., 2011).
ii) ApoCII is an LPL cofactor (Wolska et al., 2017). People bearing mutations in one of these genes (LPL, ApoAV, or ApoCII) are affected by hypertriglyceridemia: since they are not able to efficiently distribute TG to tissues, TG accumulates in the bloodstream (Carrasquilla et al., 2021).
iii) ApoE, used by lipoproteins to access target cells, is discussed in the next paragraph.

The final players involved in the TG release process are angiopoietin-like proteins (ANGPTLs) 3, 4, and 8. They are expressed differentially according to the fasting or fed state, and they function as inhibitors of LPL activity (Zhang, 2016). Loss-of-function (LOF) mutations in ANGPTL3 in humans lead to hypotriglyceridemia (Kathiresan et al., 2008) and to unexplained reductions in LDL levels. ANGPTL3 is described in the following section as a possible target for lipid-lowering drugs (see Figure 1A).

#### CM remnant uptake by hepatocytes

Deprived of most TG, CMs turn into CM remnants (75 nm) (Cooper, 1997). ApoCII is given back to HDL (Wolska et al., 2017). The reduced size of CM remnants, compared to CM, allows them to be “sleeved” through endothelial hepatic *fenestrae* and to reach the space of Disse in the liver (see Figure 1A). There, CM remnants are rapidly internalized by hepatocytes, based on the interactions between ApoE and LDL receptor (LDL-R) and LDL-R family members (LDL-R FMs, among which LDL-R-related protein 1, LRP1, is the most expressed by hepatocytes) (van de Sluis et al., 2017). ApoE is responsible for the clearance of CM remnants through receptor-mediated endocytosis (Mehta and Shapiro, 2022). CM remnant components are used by hepatocytes. Particularly, PL can be used for cellular membranes and proteins for amino acids, and leftover TG can be stored. Finally, cholesterol can be i) converted into bile salts, essential for the emulsification of fats assumed through diet; ii) stored as CE; iii) used for cellular membranes; and iv) re-packaged into very low-density lipoproteins (VLDLs) (Trapani et al., 2012). Concerning bile salts, their classic synthesis starts with 7 alpha-hydroxylation of cholesterol and converges in the secretion of bile salts into the bile canaliculus through the bile salt export pump (Trapani et al., 2012). However, not all cholesterol ends up in the bile canaliculus in the form of bile salts, since the aforementioned ABCG5 and ABCG8 are expressed even by hepatocytes (Yu et al., 2002b). ABCG5 and ABCG8 mediate the transport of free cholesterol into the canaliculus, representing a relevant route for excretion. Transgenic mice overexpressing human ABCG5 and ABCG8 show cholesterol absorption reduced by 50% and bile cholesterol levels increased >5-fold (the contribution of the hepatocytes). In a compensatory fashion, these mice increase cholesterol synthesis by hepatocytes (Yu et al., 2002b). In contrast, mice lacking ABCG5 and ABCG8 display lower biliary cholesterol amounts and 2–3-fold increases in absorption of dietary plant sterols, resembling the condition of people bearing mutations in these two genes (Yu et al., 2002a). Cholesterol in hepatocytes derives from the CEs present in lipoproteins and from *de novo* synthesis. Particularly, the expression of LDL-R, which is responsible for lipoprotein internalization, is synchronized with the synthesis of cholesterol: the less LDL-R is produced, the less cholesterol is synthesized (Horton et al., 2002). A third gene is tied directly to LDL-R: its negative modulator, proprotein convertase subtilisin kexin type 9 (PCSK9) (Abifadel et al., 2003). PCSK9 is described later, given its implication in FH and as a possible target for lipid-lowering drugs. As mentioned previously, excess cholesterol present in hepatocytes is packaged into VLDL and secreted into the bloodstream (Figure 1B).

#### VLDL formation and secretion by hepatocytes

The steps required for VLDL assembly resemble those mentioned for CM formation, with one main difference: the structural component of VLDL is ApoB100 instead of ApoB48 (Chen et al., 1987; Powell et al., 1987). In the human liver, the RNA editing enzyme APOBEC1 is not expressed; thus, the *ApoB* gene is translated into its complete form. Assembly of VLDL requires the already-mentioned MTTP (Swift et al., 2003). Again, only one molecule of ApoB is required *per* VLDL (Elovson et al., 1988; Sundaram and Yao, 2010; Sniderman et al., 2019), which contains PL, free cholesterol, CE, and TG. ApoB expression is constitutive. TGs themselves determine the rate of VLDL production, since ApoB is degraded when TGs are not sufficient to justify generation of VLDL (Nakajima et al., 2011) (see Figure 1B). Once in circulation, VLDL can either be internalized by VLDL receptor (VLDL-R), expressed in heart, muscle, and adipose tissues (Huang and Lee, 2022), or subjected to same destiny as CM. In the latter case, the following events occur: i) ApoCII and ApoE, derived from HDL, bind to VLDL; ii) LPL-ApoCII in adipose tissue and skeletal muscle capillaries mediate TG hydrolysis; iii) ApoCII moves back to HDL; and iv) VLDL turns into intermediate-density lipoproteins (IDLs) (Huang and Lee, 2022). The newly formed IDLs can be i) internalized by hepatocytes like CM remnants or ii) moved to the adrenal cortex, which expresses LDL-R and LDL-R FM (Schneider et al., 1982; Hoekstra et al., 2010; Hu et al., 2010). There, the cholesterol just internalized can be used to generate different steroid hormones, or it can be stored. IDL can also be iii) deprived of leftover TG through the action of HL in the liver and adrenal cortex (Choi et al., 1994). When this happens, ApoE moves back to HDL, and IDLs turn into LDL, which contains a very low amount of TG and is mostly composed of cholesterol and CE (Figure 1C). The newly formed LDL, being smaller than the other lipoproteins (Islam et al., 2022), circulates and moves to anatomical districts, such as gonads, which express LDL-R. There, the cholesterol contained can be used once again to make hormones, in this case, sex hormones (Choi et al., 1994). Despite the contribution of the adrenal cortex and gonads to the uptake of LDL, the vast majority of LDL (around 75%–80%) (Ishigaki et al., 2019) moves back to the liver, where it interacts with LDL-R and is internalized (Figure 1D). ApoB100, a structural component of LDL, can interact exclusively with LDL-R and with none of the LDL-R FM. This is one of the main reasons why in FH, if the receptor is mutated, the main lipoprotein massively accumulating in the bloodstream is LDL. Once inside hepatocytes, LDL components undergo the same destiny described for CM remnants. In healthy individuals, the system works as described thus far, and LDL cholesterol is kept within the normal range. However, in individuals with high LDL amounts, atherosclerosis may develop.

### Low-density lipoprotein-driven atherosclerosis

Atherosclerosis is defined as a disease of the arteries and is characterized by the deposition of fatty material, called atherosclerotic plaque, within the tunica intima of the arterial wall. For a detailed explanation of the events converging in this outcome, we refer to other, more detailed reviews (Libby et al., 2019; Libby, 2021a). Here, we briefly summarize how atherosclerosis develops in hypercholesterolemic individuals. Hypercholesterolemia may induce damage in the cells of the endothelial wall; however, the mechanisms by which LDL triggers lesions remain unclear (Libby, 2021b). Endothelial damage increases the permeability of the endothelial lining, allowing LDL to access the vessel wall. LDL accumulates in the tunica intima, where the absence of plasma antioxidants results in increased oxidized LDL (oxLDL). Moreover, dysfunctional endothelial cells expose, on the vessel surface, adhesion molecules that recruit inflammatory cells—particularly monocytes—which in turn access the sub-endothelial layer through diapedesis. There, monocytes turn into macrophages and start phagocytosing oxLDL. Macrophages filled with oxLDL are called “foam cells,” as their appearance becomes foamy. Smooth muscle cells localized in the tunica media are also involved in the formation of the plaque; in particular, they migrate from the media to the intima, where they start proliferating and synthesizing extracellular matrix, which constitutes the fibrous cap overlying the lipid core. This plaque is covered by endothelial cells. Since cells proliferate and perish during this process, the inflammatory state is increased. As the plaque progresses, the vessel lumen is reduced, thereby causing tissue damage as a consequence of decreased or absent blood flow, as in myocardial infarction. Moreover, the plaques often develop calcification. Calcified atheromas may have a higher tendency to rupture. If left untreated, this series of events may lead to plaque disruption and thrombosis (Libby, 2021b). Despite the threat represented by foam cells, humans still have a protective mechanism under healthy conditions: HDL, which can counteract the development of atherosclerosis by inducing cholesterol efflux from foam cells.

### Reverse-cholesterol transport: HDL

The initial component of the HDL particle, apolipoprotein A1 (ApoA1), is expressed mainly in enterocytes and hepatocytes. ApoA1 circulates in the bloodstream and interacts with the receptors ATP-binding cassette protein A1 (ABCA1) and ATP-binding cassette protein G1 (ABCG1), which are expressed on tissues and macrophages (Rader and Tall, 2012). Upon interaction with ABCA1, cholesterol is released in the nascent HDL, which then moves to ABCG1, incorporating additional cholesterol. Mature HDL moves back to the liver, interacts with SRB1 present on the hepatocyte membrane, and releases the carried cholesterol (Heinecke, 2012). Now reduced in size, HDL recommences its journey into the bloodstream, while cholesterol internalized into hepatocytes can be excreted into the bile. HDL’s incorporation of excess cholesterol from tissues and macrophages, which transport it to the liver where it can be eliminated, has been named “reverse cholesterol transport.” It is supposed to play a major role in counteracting the atherosclerosis process. Indeed, mice and rabbits lacking a functional ApoA1 showed increased atherosclerosis, while *ldlr* ^*−/−*^ mice overexpressing human ApoA1 showed >50% reduction in atherosclerosis progression after 6 months of high-cholesterol diet, compared to controls (Belalcazar et al., 2003). Increasing HDL levels has been pursued as a strategy for reducing the risk of CVD (known as the “HDL cholesterol hypothesis”). However, over time, the causal relationship between higher HDL amounts and reduced CVD risk has been challenged (Voight et al., 2012). It has more recently been proposed that the most relevant parameter should be the functionality of HDL (i.e., its cholesterol efflux capacity) rather than its absolute amount (Rader and Hovingh, 2014; Hovingh et al., 2015). As mentioned previously, HDL contains multiple apolipoproteins in addition to ApoA1, like the aforementioned ApoCII and ApoE. HDL donates them to CM, VLDL, and IDL, together with part of the CE. This is carried out as a trade: CEs are given and TGs are received in exchange. The mediator of this swap is cholesterol ester transfer protein (CETP), discussed in the following section, which has been unsuccessfully selected as a lipid-lowering target in the past but has been recently revived as target (Nicholls et al., 2022).

## Familial hypercholesterolemia

### Genes involved and clinical manifestations

**FIGURE 2:**
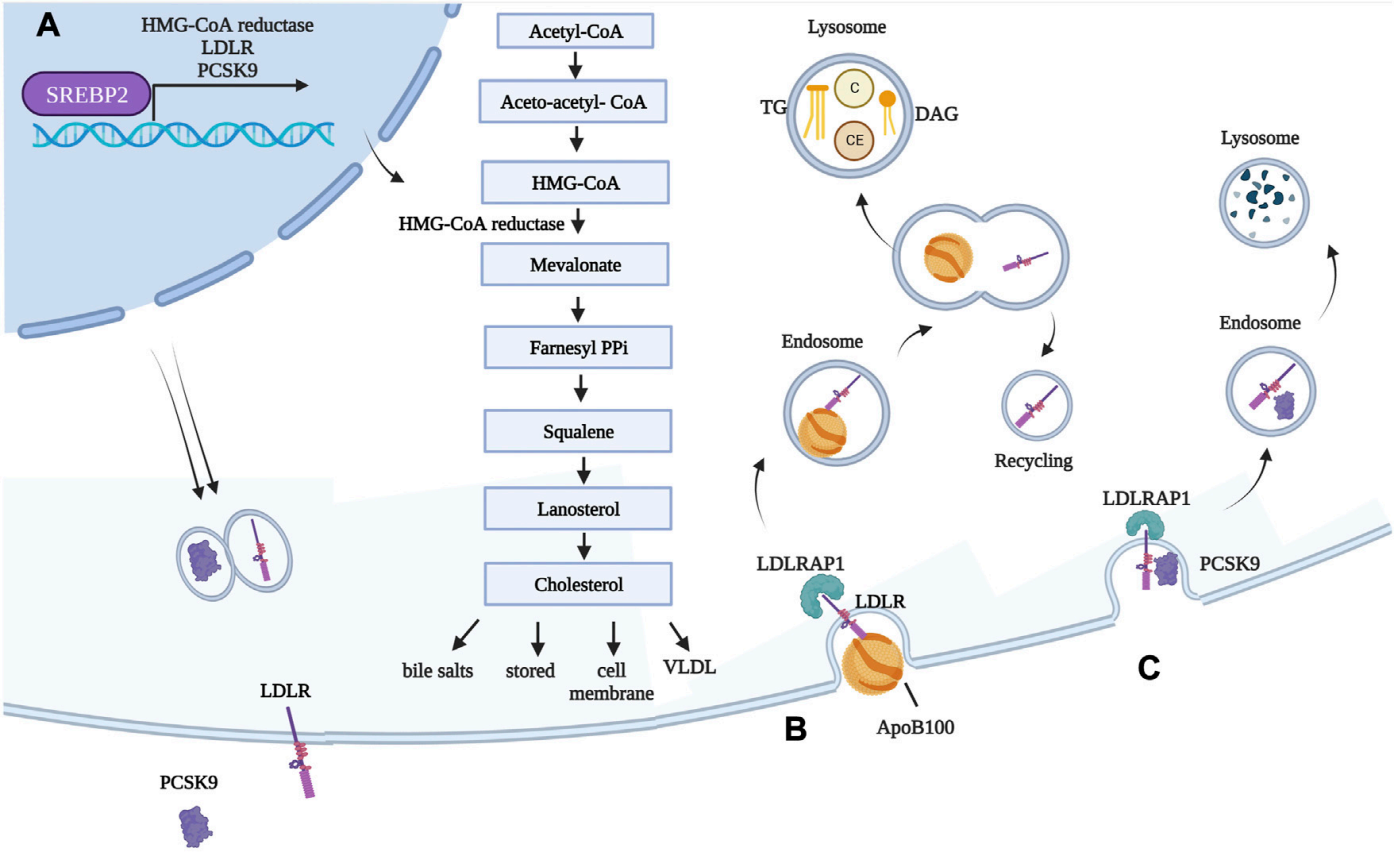
Genes involved in FH and their functions in normal conditions. **(A)** Transcription factor SREBP-2 is responsible for expression of HMG-CoA reductase, as well as LDL-R and its modulator PCSK9. Once translated, LDL-R is exposed on hepatocyte cell membranes, while PCSK9 is secreted into the circulation. HMG-CoA reductase converts HMG-CoA into mevalonate. This is the rate-limiting step during cholesterol synthesis, and the enzyme is inhibited by statins. Newly synthetized cholesterol is then i) stored as cholesterol ester, ii) used for cell membranes, iii) used for generating bile salts, and iv) packaged into VLDL. **(B)** LDL is internalized into hepatocytes (and extra-hepatic tissues) following interaction between ApoB100, a structural component of LDL, and LDL-R. LDLRAP1 mediates internalization of LDL-R, which occurs through clathrin-mediated endocytosis. The early acidic endosome environment induces disassociation of LDL-R and LDL, with the former being recycled back onto the cell membrane. As LDL-containing endosomes mature in lysosomes, LDLs are subjected to the action of lysosomal enzyme, and their components are released. **(C)** PCSK9 binds to LDL-R exposed on cell membranes. Because of the tight interaction between the two, in this case, the whole complex is targeted to the lysosome for degradation. Created with BioRender.com. C = cholesterol; CE = cholesterol esters; DAG = diacylglycerol.

FH is an autosomal dominant disease, primarily due to mutations in four genes: i) LDL-R LOF mutations, accounting for >90% of patients; ii) ApoB100 LOF mutations (Innerarity et al., 1987); iii) PCSK9 gain-of-function (GOF) mutations (Abifadel et al., 2003); and iv) low-density lipoprotein receptor adaptor protein 1 (LDLRAP1) LOF mutations, the only ones leading to a recessive form (Tada et al., 2011). LDL-R, if mutated, cause FH due to massive accumulation of LDLs in the circulation, which are unable to access hepatocytes and extra-hepatic tissues. Excluding deletions and mutations affecting regulatory regions, more than 1300 missense or non-sense mutations have been reported to occur throughout the gene (https://www.hgmd.cf.ac.uk/ac/gene.php?gene=LDLR)*.* Particularly, among its 860 codons, 550 are reported to cause hypercholesterolemia if mutated. Mutations, spread throughout the protein, can lead to a receptor that is i) defective in ligand-binding; ii) incapable of transporting its ligand; iii) internalization defective; iv) unable to be recycled; or v) null, where the protein is undetectable (Hobbs et al., 1990)*.* ApoB100, instead, is the ligand required for LDL to enter through the LDL-R. Thus, if ApoB is mutated, the interaction with its receptor is less efficient and LDL accumulates in circulation, causing FH as well. PCSK9 is the modulator of LDL-R levels; indeed, it causes LDL-R degradation instead of its recycling on the cell membrane. FH-causing mutations in PCSK9 are thus GOF mutations: in patients carrying these mutations, LDL-R degradation is enhanced and its amounts on the cell membrane become insufficient to clear LDL from circulation. PCSK9 expression, as with LDL-R, is activated during cholesterol synthesis by sterol-regulatory element-binding protein 2 (SREBP-2) (Horton et al., 2002). It can interact with LDL-R both intracellularly, mediating its degradation prior to its expression on the membrane, and extracellularly since it is released into the bloodstream (Poirier et al., 2008). The portion of LDL-R interacting with PCSK9 is different from the portion interacting with ApoB contained in LDL (Wang et al., 2021). While, conventionally, LDL-R-LDL complex is internalized and the receptor recycled back multiple times, in the case of PCSK9 binding to LDL-R, the entire complex is brought to the lysosome to be degraded. Finally, the role of LDLRAP1 is to mediate the internalization of LDL-R (Table 1; Figure 2). From the clinical point of view, HeFH is more manageable, while HoFH requires multiple combined treatments, as discussed in the following sentences. The frequency of FH is 1:250 individuals for HeFH and 1:250,000–300,000 for HoFH. Ideally, circulating total cholesterol and LDL should be < 200 mg/dL (<5 mmol/L) and <100 mg/dL (<2.5 mmol/L), respectively. In heterozygous patients, they range between 350–550 mg/dL (9–14 mmol/L) and >200 mg/dL (>5 mmol/L), respectively (Pejic, 2014). Compared to normal individuals, heterozygous untreated patients have a 50% increased risk of CVD by age 50 for men and a 30% increased risk by age 60 for women. In homozygous patients, circulating total cholesterol and LDL range between 650–1,000 mg/dL (17–26 mmol/L) and >400 mg/dL (10 mmol/L), respectively (i.e., four times more than in healthy subjects). HoFH patients display atherosclerosis in childhood, CVD within the first two decades of life, and myocardial infarction and death before 30 years of age, if left untreated. In addition to CVD, another typical manifestation of ongoing cholesterol accumulation in the wrong body compartments is the presence of xanthomas and xanthelasma. Both terms refer to the cutaneous manifestation of lipidosis, the first due to foam cells within the skin and the second at the level of the medial portion of the eye (Tromp et al., 2022).

**TABLE 1:** Genes mutated in FH and descriptions of their physiological roles.

Gene	Function	Mutation	Effect
*LDL-R*	Apolipoprotein receptor	LOF	Lack of receptor on the cell surface impedes LDL access. LDLs accumulate in the bloodstream
*APOB100*	Structural component of VLDL, IDL, and LDL	LOF	ApoB-LDL-R interaction is impaired. LDLs accumulate in the bloodstream
*PCSK9*	Protease inducing LDL-R degradation	GOF	Increased LDL-R degradation. Reduced receptor exposed on the cell membrane. LDLs accumulate in the bloodstream
*LDLRAP1*	LDL-R internalization	LOF	Impairment in LDL-LDL-R internalization

### Available treatments for familial hypercholesterolemia

#### Liver transplantation

Historically, the only available option when FH was discovered was liver transplantation. Despite being a lifesaving alternative for children affected by FH, it was not sufficient to reach circulating LDL levels comparable to those of healthy people (Starzl et al., 1984) because around 75%–80% of LDLs are captured by hepatocytes through LDL-R, as mentioned previously. Since the rest of the body remains devoid of LDL-R, the contribution of extrahepatic tissues remains absent. Notwithstanding this aspect, LDL amounts are decreased almost by 80% following liver transplantation; however, all the consequences of a transplant persist (i.e., surgical complications and the requirement of lifelong immunosuppression). Moreover, liver transplantation remains a high-risk procedure, although the outcomes are generally favorable. Whatever the treatment for a patient affected by HoFH, the sooner the treatment is delivered, the better it is. Notably, of >40 liver transplants performed from 1984 in HoFH patients, 10 were concurrent liver and heart transplantations since the development of heart disease was already exaggerated. Given the paucity of available donors for liver transplantation, an alternative deserving mention is the liver transplantation of homozygous patients using their heterozygous parents as donors, recently reported to be successful in Japan (Ishigaki et al., 2019). This kind of procedure, attempted for the cure of HoFH, has been the first case of liver transplant from a living donor.

#### Low-density lipoprotein apheresis

Despite being a valuable option for reducing LDL, which in this case would be cleared from the body, this extracorporeal technique is not devoid of issues, including high costs and lifelong maintenance of blood access. The duration of each session of LDL apheresis is ∼140 min, and the process must be repeated every 2 weeks. Despite the name, other plasma components are also removed from the blood of patients, including PCSK9, oxLDL, and inflammatory cytokines, which may be a beneficial aspect. Once again, the sooner the better, and homozygous patients starting the procedure in adulthood have poor prognoses (Makino et al., 2019).

#### Statins

Statins inhibit the enzyme HMG-CoA reductase (see Figure 2), which catalyzes the oxidoreduction of HMG-CoA to mevalonate during cholesterol synthesis. They are classified as having high intensity or moderate intensity. Statins are most often administered orally on a daily basis. Their capability to reduce LDL content is due to i) reduced intra-cellular synthesis of cholesterol and ii) increased expression and exposure of LDL-R on the membrane of hepatocytes, which in turn interact with LDL, mediating their internalization. This happens because once the hepatocyte senses that the cholesterol inside the cell is not enough, it increases its synthesis as a feedback mechanism. The transcription factor mediating cholesterol synthesis, SREBP-2, however, also mediates expression of the LDL-R (Horton et al., 2002). Thus, cholesterol levels are kept down, despite increased activation of the cholesterol biosynthetic pathway because HMG-CoA reductase is inhibited while the amounts of LDL-R increase over time. This, in turn, mediates the internalization of more LDL and the release of cholesterol in the cell (Wang et al., 2021). The first statin to be approved was lovastatin in 1989, followed by pravastatin and simvastatin in 1991. The Scandinavian Simvastatin Survival Study (4S) showed that statins were effective even as a strategy for secondary preventions: participants had experienced heart attack or angina, and deaths were reduced in the treated group compared to controls (8% deaths vs 12% deaths) (Pedersen et al., 2004). Over time, it emerged that i) statins had benefits for both primary (Shepherd et al., 1995) and secondary CVD prevention; ii) more intensive statins were better than less intensive dosing (Cannon et al., 2004) (Nissen et al., 2006); iii) the greater the reduction in LDL cholesterol, the greater the reduction in cardiovascular events; and iv) not all statins were equally safe, as cerivastatin was withdrawn in 2001 after 52 deaths caused by kidney failure following drug-related rhabdomyolysis (Furberg and Pitt, 2001). Statins have revolutionized the management of hypercholesterolemia patients. However, while the reduction in HeFH was up to 60%, HoFH reduction could barely reach 20%. Indeed, since the vast majority of HoFH patients lack a functional LDL-R, circulating LDL remains unable to access liver and extra-hepatic tissues. The most common side effect of statins is skeletal muscle pain, observed in 10%–15% of patients (Adhyaru and Jacobson, 2018); however, this is a controversial topic (Cholesterol Treatment Trialists, 2022).

#### Bile acid sequestrants

 Bile acid sequestrants inhibit the reabsorption of bile salts by enterocytes. Bile salts are synthetized from cholesterol, in the liver. If bile salts are eliminated from the body, hepatocytes induce a feedback loop to restore bile salt amounts, causing more cholesterol to be pushed out of the hepatocyte in the canaliculus (Feng et al., 2021). The available sequestrants include colesevelam (15% reduction), colestipol (16% reduction), and cholestiramine (10% reduction). As mentioned previously, HoFH patients in most cases lack a functional LDL-R. Thus, the cholesterol accessing hepatocytes is considerably reduced. Indeed, hepatocytes synthetize more cholesterol in HoFH patients than in healthy individuals. If the amount of cholesterol excreted by hepatocytes is further increased by the use of bile acids sequestrants, their response will be a further increase in cholesterol synthesis. This is why the option of bile acid sequestrants is not effective in HoFH (Sniderman et al., 2013).

#### NPC1L1 as a target

NPC1L1 is responsible for cholesterol transport from the intestinal lumen inside enterocytes and for cholesterol reuptake from the bile canaliculus. Mouse *npc1l1* knockouts are resistant to atherosclerosis even when fed a high-fat, high-cholesterol diet (referred to as the Western diet), despite an *ldlr* ^−/−^ or *apoE* ^−/−^ genetic background (Davis et al., 2007) (Yamamoto et al., 2019). Humans carrying homozygous LOF mutations in NPC1L1 have not been described to date. However, heterozygous individuals display a mean LDL reduction of 12 mg/dL (0.3 mmol/L), which has been reported to reduce the risk of CVD by >50% (Myocardial Infarction Genetics Consortium et al., 2014). This is because the reduction, while minimal, is lifelong. This evidence suggests NPC1L1 to be a good target for lipid-lowering purposes. Zetia (ezetimibe) is a selective and highly potent inhibitor of cholesterol absorption since it acts by inhibiting NPC1L1 (Van Heek et al., 1997; Bays et al., 2001). NPC1L1 is expressed by both enterocytes and hepatocytes in humans. Ezetimibe acts on both cell types, leading to both reduced cholesterol absorption and decreased reuptake from the bile canaliculus, thus lowering plasma cholesterol. Its efficacy is limited in HoFH patients.

#### PCSK9 as a target

In recent years, PCSK9 has been selected as a target to be therapeutically inhibited in hypercholesterolemia. PCSK9 GOF mutations were discovered to be associated with high cholesterol in 2003 (Abifadel et al., 2003)*,* when a French proband, aged 49, died from myocardial infarction. His LDL was 356 mg/dL (9 mmol/L), but his LDL-R and ApoB did not carry mutations. The GOF mutation F216L in PCSK9 was associated with the cause of death. Following this evidence, other groups looked deeply into their hypercholesterolemic cohorts, and PCSK9 was confirmed as mutated and causing protein GOF in some of these people. PCSK9 overexpression in wild-type mice, through adenoviral (AdV) vector-mediated delivery of PCSK9 cDNA, made the mice hypercholesterolemic (Maxwell and Breslow, 2004), while the generation and characterization of PCSK9 knockout mice showed that they were hypocholesterolemic (Rashid et al., 2005). A parabiotic experiment joining wild-type and PCSK9-overexpressing mice showed decreased LDL-R in the livers of the wild-type mice, making clear that the secreted form could induce hypercholesterolemia by inhibiting the LDL-R (Lagace et al., 2006). In the same period, healthy people carrying LOF mutations in PCSK9 were identified. They displayed hypocholesterolemia, around 30% lower circulating LDL amounts (Cohen et al., 2005; Cohen et al., 2006), and up to an 88% reduction in the risk of developing CVD. Taken together, this knowledge of the positive correlation between high PCSK9 and high LDL levels opened the way to targeting PCSK9 for reducing LDL. The first strategy to be developed was based on a neutralizing monoclonal antibody (mAb) (Chan et al., 2009)*.* At that point, it seemed that this strategy could be applied only to patients with one functional copy of LDL-R (HeFH) since the mAb was not effective in LDL-R KO mice (Chan et al., 2009). The first anti-PCSK9 mAb tested in humans clarified i) that LDL levels could be significantly reduced in healthy individuals, as well as hypercholesterolemic and genetically confirmed HeFH patients; ii) that the effect was significant even in patients concomitantly taking statins; and iii) that the effect, in combination with statins, was additive. The reason for the additive effect is that statins, by inducing the activity of SREBP-2, enhance the production of LDL-R, while PCSK9 mAb decreases its degradation (Stein et al., 2012). These positive results led to the approval of the first mAb targeting PCSK9, alirocumab (Praluent, Sanofi-Regeneron), in 2015, followed by evolocumab (Repatha, Amgen) in the same year (Sabatine et al., 2017). Currently, alirocumab is taken subcutaneously every 2 weeks (Robinson et al., 2015). Evolocumab administration is subcutaneous every 2 weeks, or once monthly at a higher dose (Sabatine et al., 2017). The outcome for LDL is comparable between the two: a reduction of around 60% in HeFH patients (Raal et al., 2014). Surprisingly, when alirocumab was tested in HoFH patients (once every 2 weeks), a 26.9% decrease in LDL levels was obtained (Blom et al., 2020). Similar results have been obtained using evolocumab for HoFH, with a follow-up of over 1 year. Injections every 4 weeks showed stable LDL reductions of 54.9% in HeFH and 21.2% in HoFH (Santos et al., 2020). The partial efficacy is probably due to residual activity of the LDL-R. Based on these promising results, inhibiting this target directly at the RNA level seemed an attractive alternative. PCSK9 was efficiently silenced in mice, rats, and cynomolgus monkeys (Frank-Kamenetsky et al., 2008) following the injection of a lipid nanoparticle (LNP) carrying a small interfering RNA (siRNA). LDL reduction was 50%–60%, and the effect lasted for 3 weeks. The drug was then optimized through an *escamotage* series that turned it into inclisiran. First, its structural component was switched from LNP to N-acetyl-galactosamine (GAlNac); this trick made it subcutaneously injectable, with a high hepatocyte-targeting efficiency, since GAlNac exploits the asialoglycoprotein receptor (ASGPR) abundantly expressed on the membrane. Subsequent chemical modification of the siRNA improved its molecular stability and activity (Fitzgerald et al., 2014). Efficacy was confirmed in phase I (Fitzgerald et al., 2017) and phase II (Ray et al., 2017) clinical trials. Inclisiran is now approved and is currently the most promising tool available in terms of efficacy, safety, and convenience for the treatment of HeFH. Patients receive a first dose, followed by the second dose 3 months later, and the subsequent administration is every 6 months. The drug is provided in a prefilled syringe containing inclisiran (commercially named Levquio).

#### ApoB100 as a target

FH occurs when mutations in this gene are downstream of the first 48% of the protein (Innerarity et al., 1987). Mutations in the upstream portion of ApoB do not result in reduced internalization of LDL in hepatocytes, giving rise to FH; rather, they result in inefficient synthesis of CM in the intestine. Since ApoB48 is a structural component of CM, mutations in this portion result in apobetalipoproteinemia, a disorder arising from intestinal malabsorption (Lee and Hegele, 2014). When FH is due to ApoB100, the amino acid residues interacting with LDL-R are the ones mutated. ApoB has been selected as a possible target to lower cholesterol in FH patients, with the idea of inhibiting VLDL formation in the liver, thus allowing a reduction of circulating cholesterol. Mipomersen is a second-generation anti-sense oligonucleotide (ASO) targeting the ApoB gene’s RNA. Mouse studies using ISIS-1467764, which targets the murine counterpart, have shown it to reduce circulating ApoB, and thus LDL, following injections in both healthy and *ldlr* ^*−/−*^ mice (Mullick et al., 2011). Preclinical studies in animal models provided evidence of reductions in ApoB, total cholesterol, and LDL in a dose-dependent manner. During clinical development, the phase III clinical trial showed an additive effect of mipomersen in HoFH when combined with statins, with an LDL reduction of 27% (Raal et al., 2010). However, several side effects have been reported, among which the most concerning is liver steatosis, due to the accumulation of TG and cholesterol inside hepatocytes since the formation of VLDL is impeded. Steatosis is resolved once the treatment is discontinued. In the end, the drug was approved exclusively in the United States, where the Food and Drug Agency (FDA) authorized its use for HoFH patients, in combination with other therapies. The delivery is subcutaneous, once per week. Lomitapide, an inhibitor of MTTP, is a different drug targeting the same pathway (Cuchel et al., 2013). Since it impairs the formation of both VLDL and CM—unlike mipomersen, which is active only on ApoB100 and not on ApoB48 mRNA—adverse gastrointestinal events have been reported. Steatosis is also among the risks of lomitapide but to a lower extent, probably because the uptake of CM remnants by hepatocytes is reduced, making the liver less stressed and the enterocytes more stressed. Notwithstanding its side effects, it appeared safer than mipomersen and was approved by both the FDA and the European Medicine Agency (EMA). It is orally taken, under the name Lojuxta (Raper et al., 2015; Alonso et al., 2019).

#### ANGPTL3 as a target

In 2002, ANGPTL3 was hypothesized as a possible cholesterol- and TG-lowering target in a strain of mice displaying hypolipidemia (Koishi et al., 2002). Adenoviral gene transfer of ANGPTL3 and recombinant ANGPTL3 injections, which both induced a rise in plasma lipid levels, confirmed this conjecture. It acts by inhibiting the action of LPL and endothelial lipases, thus enriching the pool of TG in the bloodstream (Shimizugawa et al., 2002). ANGPTL3 was considered a good target, since people carrying mutations in this gene are hypocholesterolemic and healthy, display lower TG, lower HDL, and LDL than normal, and have reduced risk of CVD (Musunuru et al., 2010; Pisciotta et al., 2012). Heterozygous individuals have ANGPTL3 circulating levels around 50% of normal (Dewey et al., 2017). This target has been selected for treatment of hypercholesterolemia. As for PCSK9, the first treatment to be tested and approved was a mAb, followed by siRNA. The mAb was shown to decrease TG and LDL, even in mice lacking a functional LDL-R, suggesting its action to be independent of the LDL-R pathway (Wang et al., 2015)*.* The mAb evinacumab (Evkeeza, Regeneron) was approved as an add-on treatment for patients affected by HoFH and aged 12 years or older. Evinacumab is a fully human mAb administered intravenously, once monthly. The reason for its LDL reduction is still to be determined. As with PCSK9, attempts were made to target ANGPTL3 at the RNA level. As with PCSK9, even ANGPTL3-targeting ASO exploits ASGPR present on hepatocytes, by virtue of being conjugated to GAlNac. It has been developed by Ionis Pharmaceuticals, under the name Vupanorsen. Studies on several mouse models, including *ldlr* ^−/−^, showed the efficacy of this strategy, as did the first clinical trial in healthy individuals and patients with elevated cholesterol and TG (Graham et al., 2017)*.* Subsequently, a phase II clinical trial was carried out in patients with HoFH and other types of dyslipidemia. Results of this trial confirmed the reduction of ANGPTL3, up to 95% at the highest dose tested. TGs were reduced, as expected, at around 50%. LDL reduction, however, was mild and reached no more than 15%. Moreover, a dramatic increase in hepatic steatosis led to the discontinuation of the program (Bergmark et al., 2022). Thus, currently only the anti-ANGPTL3 mAb evinacumab is commercially available.

#### CETP as a target

As mentioned previously, CETP is responsible for the transfer of cholesterol and TG among lipoproteins. Specifically, it transfers cholesterol from HDL to VLDL and LDL and transfers TG from VLDL and LDL to HDL. Over the years, according to the “HDL hypothesis,” CETP inhibition was supposed to reduce LDL cholesterol. Indeed, if cholesterol transport from HDL to LDL were impeded, cholesterol could have been cleared more easily, without needing LDL-R. Moreover, mice naturally lack the *CETP* gene, and their LDL amounts in an *ldlr* ^−/−^ genetic background are considerably lower than the LDL amounts of HoFH patients. CETP is produced by the liver and secreted into the bloodstream, where it exerts its activity. CETP is supposed to perform these steps: i) it bridges between smaller HDL and larger LDL (or VLDL, or CM); ii) the two distal CETP domains twist, opening a tunnel for lipid exchange; and iii) transport of CE from HDL to LDL and TG from LDL to HDL occurs (Zhang et al., 2012). Four CETP inhibitor candidates were selected in the past three decades. A summary of the results obtained follows. Torcetrapib testing was discontinued in 2006: the trial was blocked prematurely following numerous deaths and increased CVD in the CETP inhibitor group (Barter et al., 2007). Dalcetrapib development was halted when phase III failed to show a clinical benefit; the same was true for evacetrapib, which was discontinued in 2015 (Tall and Rader, 2018). Anacetrapib was the only treatment showing modest efficacy. Compared to its forerunners, anacetrapib was safer than torcetrapib and more potent than dalcetrapib; the study was designed with a sufficiently long duration to reveal the benefit of the therapy (in contrast with evacetrapib). However, the benefit was too modest, and the sponsor (Merck) discontinued the program. Recently, CETP has come back as a possible target for lowering LDL cholesterol: obicetrapib, designed to inhibit it and with the primary objective of reducing LDL, proved to be effective in a phase IIb trial, with a 51% reduction of LDL cholesterol in patients already under a statin regimen (Nicholls et al., 2022). In principle, however, a possible side effect could be the accumulation of TG in LDL and VLDL.

## Gene therapy for familial hypercholesterolemia

### Viral vector-mediated gene transfer

In recent years, gene therapy has emerged as a promising therapeutic option for monogenic diseases, as witnessed by the ever-growing number of clinical trials and approved drug products (Iglesias-Lopez et al., 2021). Gene therapy offers the attractive prospect of being a “one-and-done” treatment by inducing a permanent genetic modification in the host for a therapeutic purpose. Indeed, with a single administration, gene therapy promises to become a potentially lifelong cure, acting at the DNA level, unlike more traditional pharmacological approaches requiring repeated drug administrations and which target downstream components (Naldini, 2015; Dunbar et al., 2018). Specifically, gene therapy can be performed by transferring a functional copy of the gene mutated in a genetic disease, for example, by a replication-defective virus referred to as a viral vector. Overall, this procedure can be referred to as “gene transfer” or “gene addition” (note that we refer to them interchangeably throughout the text). Being replication-defective, viral vectors perform only the first round of infection, referred to as transduction. *In vivo* viral vector-mediated gene transfer has been applied in preclinical models and in humans to treat congenital blindness targeting the retina, neuromuscular diseases, neurons or muscles, hemophilia, and other metabolic disorders targeting the liver (Dunbar et al., 2018; Cantore et al., 2021). Non-viral delivery vehicles can also be used for gene transfer and addition; however, viral vectors are those most commonly exploited, particularly in the context of genetic diseases. Three main viral vectors have been applied in *in vivo* gene therapy over the years: AdV vectors, adeno-associated viral (AAV) vectors, and lentiviral vectors (LVs). The main features of each of them are summarized in Table 2. Today, the most advanced strategy to transfer genes to the liver is based on direct *in vivo* delivery of AAV vectors (High and Roncarolo, 2019). A single intravenous administration of these vectors has reached late-stage clinical development and has even come to market in the context of the coagulation disorder hemophilia, showing promising safety and efficacy (Ozelo et al., 2022; Mahlangu et al., 2023; Pipe et al., 2023).

**TABLE 2:** Main viral vectors used for *in vivo* gene transfer.

	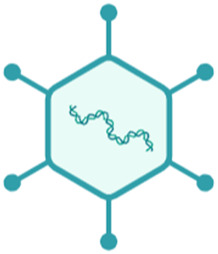	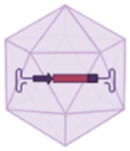	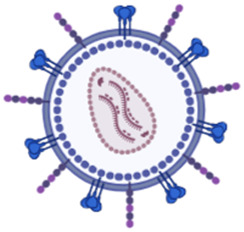
	AdV vector	AAV vector	LV
Size	90–100 nm	25 nm	120 nm
Genome	dsDNA	ssDNA	ssRNA
Cargo capacity	8–36 Kb	4.7 Kb	9 Kb
Transduction	Dividing–non-dividing cells	Dividing–non-dividing cells	Dividing–non-dividing cells
Integration	Non-integrating	Non-integrating	Integrating
Pre-existing immunity	Serotype-dependent	Serotype-dependent	Low

### Genome editing

Gene therapies also encompass genome editing strategies. The concept of genome editing defines strategies to precisely and exclusively target the desired *loci* in the genome. This outcome can be achieved using an editing machinery able to specifically recognize and cut the DNA target sequence; consequently, the broken double DNA strand can be repaired by non-homologous end joining (NHEJ), which is error prone and exploitable for gene disruption, or through homology-directed repair (HDR), which is useful if the aim is gene correction. In the latter case, the template sequence to be used for HDR is part of the editing machinery to be delivered (donor DNA). Concerning the recognition and cutting of the target DNA sequence, different tools have been developed: zinc finger nucleases, transcription activator-like effector nucleases, and clustered regulatory interspaced short palindromic repeats (CRISPR)/CRISPR-associated (Cas) nuclease. CRISPR/Cas is a dual system composed of Cas protein and guide RNA (gRNA). Cas9 performs a DNA double-strand break following gRNA binding to the target DNA site. It is currently the most widely used tool for editing the genome and earned the Nobel Prize for the scientists first describing it, Jennifer Doudna and Emilie Charpentier (Doudna and Charpentier, 2014; Doudna, 2020). No matter the technology used, once a break is introduced, the cell will fix it. Repair can occur through NHEJ or HDR, as mentioned. NHEJ is an excellent way of knocking out genes since the outcomes are frameshift mutations (indels) leading to loss of gene expression in most cases. HDR is a less efficient but accurate DNA repair pathway. The cell conventionally uses the sister chromatid to perform this process. In genome editing, the template used in place of the sister chromatid is called “donor DNA.” If the aim is gene correction through HDR, then efficiency is usually quite low and an important percentage of the cut will be fixed anyway through NEJM (Musunuru, 2022). Currently, the CRISPR/Cas9 system search-and-bind activities are being applied for new purposes: chemical modifications of DNA bases (base editing), modifications in gene expression (epigenome editing) (Amabile et al., 2016), and the introduction of new bases through the reverse transcription of an RNA used as a template (prime-editing) (Anzalone et al., 2020). Concerning FH, the most promising results using these new technologies have been achieved so far with base editing. Base editors can induce a point mutation at a specific genomic sequence. In particular, cytidine base editors (CBEs) and adenine base editors (ABEs) have been generated. In CBEs, gRNA and Cas9 search for and bind to the target sequence but make a single-stranded cut (the nickase Cas9 is used instead of standard Cas9). Fused with Cas9 is a deaminase domain (in most cases derived from Apobec). Complementarity between gRNA and the target strand induces the exposure of non-target DNA to the deaminase action, which can modify virtually every C within the editing window by turning it into a U. Uracil deaminase should revert the mutation to C, but fused to the editing machinery is uracil deaminase inhibitor (UGI), which prevents this from occurring. The nick on the target strand induces the cells to use the non-target, non-base-edited strand as a template. Thus, the U serves as a template for an A in the target strand. Ultimately, the U is turned into a T to restore Watson–Crick base pairing. To generate ABEs, protein evolution has been used to obtain novel DNA deaminases. The process is similar to CBE. In this case, adenine is turned into inosine. The complementary base introduced to fill the target strand following the nick is cytosine, and inosine is ultimately converted into guanine. Since ABEs currently seem more efficient and specific than CBEs, they are preferred when possible (Musunuru, 2022). Viral or non-viral delivery systems can be used to deliver the editing machinery and the donor DNA template, if necessary, to target tissues.

### LDL-R gene addition

As mentioned previously, LDLs accumulate in the bloodstream in cases of mutations in LDL-R. The other lipoproteins are approximately in the normal range, since they can interact with LDL-R FM in addition to LDL-R. As mentioned previously, the peculiarity of LDL is the exclusive presence of ApoB100 as a ligand for its internalization. Thus LDLs carrying ApoB100 as the only apolipoprotein can no longer access hepatocytes once the receptor is not exposed on the membrane. Even in HeFH patients, the amount of LDL-R is not sufficient to ensure normal LDL and cholesterol levels, and pharmacological intervention is required (gene dosage effect). LDL-R gene transfer, as a strategy, could be applied to LDL-R LOF mutations, to PCSK9 GOF mutations, and to the portion of mutations in ApoB responsible for a reduced interaction between LDL and LDL-R, since higher amounts of the receptor on hepatocyte membranes could ameliorate the pathological outcome.

#### First GT approach: *Ex vivo* hepatocyte-based

In the 1990s, LDL-R gene addition was attempted in hepatocytes collected from hypercholesterolemic Watanabe heritable hyperlipidemic rabbits (WHHRs). Twelve WHHRs were subjected to 30% hepatectomy. Isolated hepatocytes were transduced *in vitro* with vectors derived from retroviruses, the only vectors available at that time, and then transplanted back into the rabbits. This treatment provided a stable reduction, between 20% and 40%, of circulating cholesterol, despite the low number of transplanted cells (Chowdhury et al., 1991). Rabbits were followed for 4.5 months. In non-human primates (NHPs), the hepatocytes were confirmed to engraft following injection through the inferior mesenteric vein (Grossman et al., 1992). Following these results, a clinical trial started. Five hypercholesterolemic patients were subjected to partial hepatectomy. The procedure was the same: their hepatocytes were transduced with a retroviral vector expressing human LDL-R and then re-infused. High variability was observed among patients, with one patient having unchanged LDL levels following transplant and the best-responding patient showing a 20% LDL reduction (Grossman et al., 1995). This is among the first reports of human gene therapy in which the therapeutic endpoint was achieved in a portion of subjects. However, this strategy was not further pursued as it became clear, in the following years, that *in vivo* delivery of LDL-R transgene to hepatocytes would have been preferable, especially because it would not have required hepatectomy and would have been relatively easier to deploy.

#### *In vivo* LDL-R gene addition to hepatocytes: AdV vectors

Toward this goal, the first vectors to be used were AdV vectors. Indeed, in work describing the generation of *ldlr* ^*−/−*^ mice, an intravenous injection of LDL-R encoding the AdV vector induced a transient transgene expression, as shown by examining the livers of treated mice 4 days after infusion (Ishibashi et al., 1993). The highly immunogenic feature of AdV vector was confirmed in subsequent studies in both mice and rabbits, showing transient cholesterol reduction. Three weeks after gene therapy, cholesterol amounts were comparable to pre-treatment levels (Kozarsky et al., 1994; Li et al., 1995). A transient 50% decrease in cholesterol circulating amounts, again lasting for just 3 weeks, was achieved with a new gene therapy approach: the VLDL-receptor liver gene transfer (Kobayashi et al., 1996). As mentioned previously, the main anatomical districts where VLDL-R is normally expressed are adipose tissue, skeletal muscle, and heart. In the liver, its expression is almost undetectable. Nevertheless, forcing its expression by hepatocytes was confirmed to be a valuable strategy in the following years, when long-term cholesterol reduction was finally achieved (Kozarsky et al., 1996). The study crucially proved that, since VLDL receptor, unlike LDL-R, was already expressed by the mouse model, the risk of inducing an immune response against the transgene product was considerably lower. The mouse group treated in parallel with AdV vector expressing LDL-R, indeed, displayed only transient cholesterol reduction (21 days). Despite these promising results, while >80% of hepatocytes were VLDL-R positive at day 21, around 10% were still corrected 3 months later. AdV vectors were subsequently modified to reduce their immunogenicity and improve their safety profile by providing *in trans,* using a helper virus, the viral proteins required for vector production. With these improved vectors, VLDLR gene transfer was confirmed as a therapeutic strategy, ensuring lifelong correction of adult-treated *ldlr* ^*−/−*^ mice (Oka et al., 2001). Despite being an elegant strategy to overcome possible immune responses against LDL-R, the main limitation of this approach may be an increase in intracellular TG in the liver. Indeed, VLDL-R expression by the liver seems to correlate with liver steatosis (Jo et al., 2013). Recently, an interesting new gene therapy strategy has been described: AdV vector-mediated gene transfer of a chimeric LDL-R-transferrin transgene carrying the LDL-binding domain of LDL-R but lacking the cytoplasmic LDL-R tail, which is replaced by a transferrin dimer. This modification allows the LDL-R to be secreted into the bloodstream, bind to LDL, and be reuptaken by cells expressing transferrin receptor. This strategy allows LDL to localize to the heart and intestine, in addition to the liver; however, it did not recapitulate the physiological LDL distribution (Leggiero et al., 2019). Even in its improved versions, AdV vectors remain highly pro-inflammatory, and this feature currently challenges their use as vectors for *in vivo* gene transfer by systemic administration (Piccolo and Brunetti-Pierri, 2014). Rather, their immunogenic properties make them attractive vehicles for *in situ* delivery in cancer immunotherapy and vaccination.

#### *In vivo* LDL-R gene addition in hepatocytes: AAV vectors

As mentioned previously, among viral vectors, AAV vectors currently represent the most advanced tools for *in vivo* gene transfer to the liver and have been considered a valuable option for FH gene therapy. Efficacy and safety were demonstrated, following AAV serotype 8- (AAV8-) mediated LDL-R gene addition, in different mouse models followed long-term, including both canonical HoFH mice (*ldlr* ^−/−^) and the double-knockout mouse model *ldlr* ^−/−^ and *Apobec* ^−/−^. Indeed, compared to humans, mice express *Apobec* even in hepatocytes; thus, a considerable fraction of LDL carries ApoB48 as a structural component and can be cleared even by LDL-R FM. As a result, the double knockout model better recapitulates the high LDL cholesterol of human HoFH patients; indeed, these mice display higher LDL than do *ldlr* ^−/−^ mice thanks to the presence of exclusively ApoB100 in LDL (and CM), compared to *ldlr* ^−/−^ (and wild-type mice) (Greig et al., 2017b). The safety of the vector was then assessed in NHP, both wild type and heterozygous, for the spontaneous truncating LDL-R mutation W284stop (NHPs homozygous for this LDL-R mutation are not vital). They were sacrificed either 28 days or 1 year after gene transfer. While the safety profile was positive, the authors did not report any cholesterol reduction or evidence of LDL-R at a protein level. They found AAV vector DNA in the liver at the end of experiment, as well as LDL-R RNA, which was reduced over the time course of the experiment (Greig et al., 2017a). Following this study, a clinical trial of the AAV-mediated gene transfer of a functional copy of human LDL-R to the liver started in 2016, sponsored by REGENXBIO (ClinicalTrials.gov Identifier: NCT04080050). Nine adult patients with HoFH were treated in three cohorts of increasing vector doses. Efficacy data are still to be reported; however, the sponsor reported the discontinuation of the clinical development (http://ir.regenxbio.com/news-releases/news-release-details/regenxbio-reports-second-quarter-2020-financial-results-and/). In the meanwhile, a second-generation AAV8 vector expressing LDL-R has been generated. The main modifications are i) the inclusion of the Kozak sequence, ii) codon optimization, iii) the addition of part of the human beta-globin gene intron, iv) the incorporation of the woodchuck hepatitis virus post-transcriptional regulatory element (WPRE) at the 3′ end of the cassette, and v) modifications of the LDL-R sequence with three GOF point mutations (L318D, K809R, and C818A) reported to reduce PCSK9 and consequently receptor degradation (Gu et al., 2013)*.* All these modifications allowed the treatment of mice with significantly lower doses of AAV vectors than before (Wang et al., 2021). As mentioned previously, cholesterol biodistribution is different between mice and humans, which may limit the translation of results from mice to humans. In fact, it is now possible to perform experiments in mice with livers carrying human hepatocytes, known as “humanized mice.” These mice (FRG mice) are immune deficient and lack fumarylacetoacetate fumarase, *Fah* ^−/−^. Because the enzyme is essential for the survival of hepatocytes, these mice die if left untreated; however, they can be compensated by providing nitisinone (NTBC). Slightly after the discontinuation of the drug, human hepatocytes are transplanted. Given the selective advantage that they have toward the murine *Fah* ^−/−^ hepatocytes, they can replace them with a percentage of chimerism up to 90 (Lisowski et al., 2014)*.* The technology was successfully applied to hypercholesterolemia (Bissig-Choisat et al., 2015). Briefly, hepatocytes from a female child with HoFH undergoing liver transplantation were transplanted into FRG mice. When fed with the Western diet, they became hypercholesterolemic. The condition was reverted by delivery of an AAV9 vector encoding human LDL-R, even though gene transfer was more efficient in residual murine hepatocytes. Despite all the advantages of this approach, the remaining major limitation is that human hepatocytes are placed in a non-physiological environment in which blood composition and signals may be markedly different. In particular, in the context of FH, the mice were not hypercholesterolemic unless challenged with the Western diet, probably because of the residual uptake of cholesterol by extrahepatic tissues, together with uptake by residual murine hepatocytes carrying functional LDL-R. AAV vectors remain mostly episomal in transduced cells; thus, they are diluted upon cell proliferation, such as during liver growth, leading to a progressive decrease in the therapeutic transgene. Effective re-dosing is currently difficult to achieve due to immune responses to the vector elicited by the first administration; research efforts are underway to make re-dosing possible (Meliani et al., 2018; Leborgne et al., 2020). This limitation currently challenges the application of AAV vector-mediated gene transfer in pediatric patients. However, young children would be the ideal target population for gene therapy in FH, since they would most benefit from an early intervention. Indeed, high LDL cholesterol since birth causes progressive atherosclerosis. Recently, a novel hypercholesterolemic mouse model was generated that carries the truncating Ldlr E208stop mutation. To advance a gene correction strategy to correct this mouse model, genome editing has been attempted. AAV8 vector-based CRISPR-Cas9 and a corrective donor DNA were delivered. Genetic correction occurred in a subset of hepatocytes, and total cholesterol and TG were reduced, even if not normalized, and atherosclerotic plaques were smaller but still present, compared to untreated mice (Zhao et al., 2020).

#### *In vivo* LDL-R gene addition to hepatocytes: LV

HIV-derived LVs integrate their genetic cargo into the host cell genome and are thus maintained upon cell replication. For this reason, they have been widely adopted to transfer genes into hematopoietic stem and progenitor cells and lymphocytes in gene therapy applications (June and Sadelain, 2018; Ferrua et al., 2019; Fumagalli et al., 2022). *In vivo* administration of LVs for liver gene transfer have also been performed, with evidence of efficacy and safety in small and large animal models of hemophilia and other diseases, including in NHPs (Cantore et al., 2015; Milani et al., 2019; Milani et al., 2022; Nicolas et al., 2022); clinical evaluation may start in the near future (Cantore and Naldini, 2021). LDL-R has been reported to be the entry route of vesicular stomatitis protein G (VSV.G) pseudotyped LV (Finkelshtein et al., 2013). The interaction of LDL-R and VSV. G during LV production has been shown to cause a substantial decrease in LV production and viral titers (Al-Allaf et al., 2019). Despite this problem, LVs were used to treat WHHR, achieving a partial reduction in circulating cholesterol (around 25%). Insertional mutagenesis remains a concern for integrating vectors. It should be noted, however, that LV-mediated liver gene transfer did not result in liver carcinogenesis, even in mice treated as newborns and genetically tumor-prone or tumor-promoted, followed for long time (over 1 year) (Cantore et al., 2015; Milani et al., 2022). Moreover, analysis of LV integrations in the liver of mice, dogs, and NHPs did not show clonal expansions or enrichment of integrations near cancer genes (Cantore et al., 2015; Milani et al., 2019; Cesana et al., 2021), thus offering reassurance for a possible clinical translation.

#### *In vivo* RNA-based delivery

More recently, intravenous delivery of exosome-based LDL-R mRNA has proved to be effective in decreasing cholesterol in *ldlr* ^−/−^ mice (Li et al., 2021). A clinical trial using this strategy has started (ClinicalTrials.gov Identifier: NCT05043181), but its results are still undisclosed. However, this approach would result in transient efficacy, and repeated mRNA administration would be required.

Likely the best therapy for HoFH patients would be the gene transfer of LDL-R that is i) long-lived and administered once during the patient life; ii) administered during infancy, since the sooner the treatment, the better the outcome; iii) targeting the liver but instructing it to work more than a normal liver, since liver transplant is not fully curative; and iv) targeting the highest possible percentage of hepatocytes to avoid excessive cholesterol- and TG-containing lipoprotein uptake from the few corrected cells. Genome editing to restore physiologic LDL-R activity could be an option; however, it should be considered that the efficiency of HDR is still relatively low compared to gene transfer and that correction of the liver, even at high efficiency, would not be curative if physiological expression were restored. Indeed, as mentioned previously, overexpressing the LDL-R in a large fraction of hepatocytes would be necessary for an effective treatment of HoFH. In addition to reducing circulating cholesterol by inducing LDL intake by hepatocytes, LDL-R gene transfer to the liver would bring about the additional advantage of suppressing cholesterol synthesis by hepatocytes: incorporation of cholesterol could indeed cause the inhibition of HMG-CoA reductase, since cholesterol synthesis would no longer be required (Brown and Goldstein, 1974). LDL-R gene addition induces constitutive expression of LDL-R. However, cases of hypocholesterolemia due to deletion of the regulatory 3′ region of the LDL-R gene have been reported, without description of any health concern (Bjornsson et al., 2021), and further justify this therapeutic strategy.

### Genetic inactivation of PCSK9 and ANGPTL3 by genome editing

Toward a “one-and-done” inactivation of PCSK9, genome editing, base editing, and epigenome editing have been exploited (Wang et al., 2018; Musunuru et al., 2021; Rothgangl et al., 2021; Kaiser, 2022), with the most advanced being base editing presently. Particularly, ABEs were used to treat 4 NHPs (Musunuru et al., 2021). mRNA encoding for ABEs and guide RNA (gRNA) targeting PCSK9 were packaged in an LNP and injected systemically by a one-time treatment. Over 60% of the liver was edited. Reductions of circulating PCSK9 and LDL were 90% and 60%, respectively, and were stable for 8 months. The follow-up study showed that the treated NHPs maintained the reduction in PCSK9 and LDL until sacrifice at 476 days and that their offspring did not inherit the modification (Lee et al., 2022). In July 2022, Verve Therapeutics, the company sponsoring these studies, administered the first base editor *in vivo* in humans. The single-dose, dose-escalating trial, based in New Zealand and the United Kingdom, aims to deliver a definitive cure for HeFH. While interim results are expected in 2023 (ClinicalTrials.gov Identifier: NCT05398029), three patients have been treated so far. Attempts to genetically inactivate ANGPTL3 have also been undertaken by Verve Therapeutics. The strategy to permanently inactivate ANGPTL3 in hepatocytes is designed to treat HoFH. However, the inefficient entry of LNP in hepatocytes lacking LDL-R brought the development of LNP exploiting the already mentioned GAlNac–ASGPR axis (Kasiewicz et al., 2021). This modification allows editing efficiencies comparable to those achieved with wild-type mice treated with standard LNP. The second *escamotage* used by Verve to increase the editing efficiency of its BE was to move from a CBE to an ABE. It is now quite accepted that CBEs are less efficient than ABEs. This is probably because CBEs induce a genomic modification that the cell is ready to correct, thanks to the presence of uracil DNA glycosylase activity. Inclusion of two tandem UGI sequences in the construct containing the CBE is likely not yet sufficient to make it as efficient as ABE. To induce a genetic inactivation using ABE, Verve disrupted a splice donor in the ANGPTL3 sequence, ultimately causing a shift in the open reading frame leading to a premature stop codon. With this configuration, they obtained almost a 90% reduction in circulating ANGPTL3, but reduction of circulating LDL cholesterol has not been reported yet (Khera et al., 2022). Verve selected this ABE as their leading candidate for HoFH. As mentioned previously, PCSK9 targeting in HoFH can induce partial reduction in LDL levels. Verve Therapeutics showed that LNP can be administered subsequently in the same NHP*.* The aim is to genetically inactivate PCSK9 and ANGPTL3 using the same LNP core. Eleven monkeys were injected with PCSK9-targeting BE, followed by ANGPTL3-targeting BE 1 month later. Fifteen days after the second injection, 69% editing of PCSK9 and 52% editing of Angpt3 were achieved in the liver. Their strategy could imply the future administration of both LNPs with the hope of achieving an additive reduction in LDL levels not shown yet (Bellinger et al., 2022).

## Conclusion and perspectives

HeFH is relatively manageable from the clinical point of view, since approximately a 2–3-fold reduction is sufficient to bring LDL cholesterol to normal levels. This goal can currently be achieved using statins, ezetimibe, and potentially PCSK9 inhibitors (by either neutralizing mAb or, more recently, siRNA). In contrast, clinical management of HoFH patients remains very challenging, since more than a five-fold reduction of LDL cholesterol is required for effective treatment. Statins, ezetimibe, bile acid sequestrants, and ApoB100-targeting drugs all have limited efficacy in HoFH. PCSK9 inhibitors are partially effective and only in patients displaying residual LDL-R activity. In addition, PCSK9 inhibition only accounts for a 20% reduction of LDL cholesterol in HoFH with residual LDL-R activity. ApoB100-targeting drugs only allow a 27% reduction of LDL cholesterol when combined with statins in HoFH. More than five treatments are reported to be necessary to fully normalize LDL cholesterol in HoFH, among which are statins, ezetimibe, ApoB100-targeting drugs, PCSK9 inhibitors, and anti-ANGPTL3 mAb, as well as LDL apheresis, which is invasive and negatively impacts quality of life (Tromp et al., 2022). As mentioned previously, liver transplantation is effective in HoFH patients, even if it does not result in full normalization of LDL cholesterol. However, it remains associated with numerous side effects and a paucity of available organs. For all the aforementioned reasons, alternative treatment options for HoFH are highly needed. If proved to be safe and effective in HeFH patients, genetic inactivation of PCSK9 (e.g., by base editing) may in the future substitute for anti-PCSK9 mAb or siRNA, which require multiple administrations to maintain efficacy. For HoFH patients, LDL-R gene transfer to the liver may be the most desirable option (Tromp et al., 2020). Since liver transplantation is not fully corrective in HoFH patients, we assume that overexpressing LDL-R in a large portion of hepatocytes would be required in a gene therapy setting. Site-specific correction of LDL-R at its endogenous locus may not be as effective as viral-vector mediated gene transfer in this case because physiological LDL-R expression may not be sufficient. Ideally, the gene transfer procedure should be performed as soon as possible after diagnosis to prevent accumulation of damage and reduce the risk of CVD. For this reason, the use of integrating vectors such as LV may be preferred, as it would allow stable LDL-R expression following a single administration and potentially lasting for the entire life of the recipient. AAV vector re-administration is currently being attempted in preclinical models. If AAV-vector-mediated LDL-R gene transfer is effective in adult patients, re-administration should then be considered since young patients are the most important patient population to target. ANGPTL3 inhibition is emerging as a partially effective treatment, even in HoFH. Interestingly, while mAb-mediated inhibition of ANGPTL3 activity reduces LDL cholesterol by about 45% without apparent side effects, anti-ANGPTL3 siRNA did not exert the same effect in HoFH patients and led to hepatic steatosis, for reasons not yet fully understood. These data may suggest that inhibiting ANGPTL3 synthesis in hepatocytes lacking LDL-R is not as effective as inhibiting its activity systemically, such as by using mAb. If future gene therapy is shown to benefit FH, then its application to non-genetic dyslipidemias may also be considered. As mentioned previously, beyond FH there is acquired hypercholesterolemia. Some conditions could trigger it as a bystander effect, including type II diabetes and aging. In type II diabetes, insulin is produced, but tissues become insensitive to it. As a result, adipose tissue and skeletal muscle are induced to undergo lipolysis to release FFA in the circulation. Cholesterol and TG levels thus increase as a bystander effect. In the context of type II diabetes or spontaneous increases in LDL cholesterol due to aging, a gene therapy intervention such as inactivation of PCSK9 or LDL-R gene transfer may become beneficial. Despite the remaining unknowns and lack of long-term safety data, genetic therapies bear the potential to revolutionize medicine and positively impact people suffering from several different diseases. In addition to efficacy and safety, widespread adoption of gene therapies in clinical practice in the future will also depend on evolution of the current regulatory and reimbursement framework and on a propensity to innovation by all the involved stakeholders, including physicians, patients, and policymakers.

## Glossary

AAV vectors: adeno-associated viral vectors
ABCA1: ATP-binding cassette protein A1
ABCG1: ATP-binding cassette protein G1
ABCG5-8: ATP-binding subfamily G members 5–8
ABE: adenine base editor
AdV vectors: adenoviral vectors
ANGPTL 3-4-8: angiopoietin-like proteins 3, 4, 8
ApoB: apolipoprotein B
APOBEC: apolipoprotein B mRNA-editing enzyme catalytic polypeptide 1
ASGPR: asialoglycoprotein receptor
ASO: anti-sense oligonucleotide
Cas: CRISPR-associated nuclease
CBE: cytidine base editor
CE: cholesterol ester
CETP: cholesterol ester transfer protein
CM: chylomicron
CMR: chylomicron remnant
CRISPR: clustered regulatory interspaced short palindromic repeats
CVD: cardiovascular disease
EMA: European Medical Agency
ER: endoplasmic reticulum
FDA: Food and Drug Agency
FFA: free fatty acid
FH: familial hypercholesterolemia
GAlNac: N-acetyl-galactosamine
gRNA: guide RNA
HDL: high-density lipoprotein
HDR: homology-directed repair
HeFH: heterozygous familial hypercholesterolemia
HL: hepatic lipases
HMG-CoA: 3-hydroxy-3-methylglutaryl coenzyme A
HoFH: homozygous familial hypercholesterolemia
HSPG: heparan sulfate proteoglycans
IDL: intermediate-density lipoprotein
LDL: low-density lipoproteins
LDL-R FM: low-density lipoprotein receptor family member
LDL-R: low-density lipoprotein receptor
LDLRAP1: low-density lipoprotein receptor-related protein 1
LIPG: endothelial lipase
LNP: lipid nanoparticle
LOF: loss of function
LPL: lipoprotein lipase
LV: lentiviral vector
mAb: monoclonal antibody
MAG: monoacylglycerol
MTTP: microsomal TG transfer protein
NHEJ: non-homologous end joining
NHP: non-human primate
NPC1L1: Niemann–Pick C1-like protein 1
oxLDL: oxidized LDL
PCSK9: proprotein convertase subtilisin/kexin 9
PL: phospholipid
PLA2: phospholipase A2
SRB1: scavenger receptor B1
SREBP-2: sterol regulatory element-binding protein 2
TG: triglyceride
VLDL: very low-density lipoprotein
VLDL-R: very low-density lipoprotein receptor
VSV.G: vesicular stomatitis glycoprotein
WHHR: Watanabe heritable hyperlipidemic rabbit
